# Auditory processing deficits in subacute stroke

**DOI:** 10.1007/s00415-024-12754-x

**Published:** 2024-12-16

**Authors:** Jawad Abdulla, Nehzat Koohi, Rahul Lakshmanan, Chandrashekar Hoskote, Menelaos Pavlou, Jason D. Warren, Chris J. D. Hardy, David J. Werring, Doris-Eva Bamiou

**Affiliations:** 1 Department of Neuro-Otology, Royal National ENT and Eastman Dental Hospitals, London, UK; 2 Department of Clinical and Movement Neurosciences, University College London, Queen Square Institute of Neurology, London, UK; 3 Comprehensive Stroke Service, National Hospital for Neurology and Neurosurgery, Queen Square, London, UK; 4 Department of Medical Imaging, Perth Children’s Hospital, Perth, Australia; 5 UWA Medical School, Centre for Neuromuscular and Neurological Disorders (Perron Institute), Nedlands, Australia; 6 Neuroradiology, National Hospital for Neurology and Neurosurgery, University College London Hospitals NHS Foundation Trust, London, UK; 7 Department of Statistical Science, University College London, London, UK; 8 Dementia Research Centre, Department of Neurodegenerative Disease, UCL Queen Square Institute of Neurology, University College London, London, UK; 9 Stroke Research Centre, Department of Brain Repair and Rehabilitation, University, College London Queen Square Institute of Neurology, London, UK; 10 The Ear Institute, University College London, London, UK; 11 National Institute for Health and Care Research, University College London Hospitals Biomedical Research Centre (Deafness and Hearing Problems Theme), London, UK

**Keywords:** Auditory processing disorder, Stroke, Hearing loss, Central auditory nervous system

## Abstract

**Background:**

Stroke is the second leading cause of disability worldwide. Stroke results in focal neurological deficit and often leads to auditory problems due to its impact on the auditory pathway. Altered connections in the auditory pathway, caused by stroke, can result in hearing difficulties ranging from impaired sound detection to altered auditory perception. A better understanding of how stroke affects these early sound processing mechanisms will provide valuable insights into stroke recovery and rehabilitation options.

**Methods:**

We recruited forty consecutive adult patients with stroke (30 males, 10 females) due to ischemic or intracerebral hemorrhage > 3 and up to 12 months after stroke (subacute stage). Brain MRIs were performed on all patients, and we calculated a central auditory nervous system stroke severity index (CANS SSI) according to number of CANS areas involved and an extended CANS definition of auditory responsive areas. All patients underwent cognitive screening assessment, basic audiological assessments, and a hierarchical central auditory processing assessment battery with the Queen Square Tests of Auditory Cognition (early perceptual processing, apperceptive processing, semantic Processing) and Gaps in Noise tests.

**Results:**

When comparing patients with auditory responsive cortical lesions and with versus without Heschl’s gyrus involvement (primary auditory cortex), patients with Heschl’s gyrus involvement exhibited worse early perceptual scores. The CANS SSI showed a significant negative correlation with early perceptual test scores.

**Conclusion:**

This study demonstrates a correlation between stroke severity, characterized by a higher number of lesions involving auditory areas in patients with subacute stroke, and worse early perceptual scores. Heschl’s gyrus involvement is associated with poorer early perceptual score.

**Supplementary Information:**

The online version contains supplementary material available at 10.1007/s00415-024-12754-x.

## Introduction

Stroke is the second leading cause of disability worldwide [1]. Stroke causes focal neurological deficits attributed to vascular injury of the central nervous system. It often results in auditory deficits due to involvement of the auditory pathway [2, 3]. Both ischemic stroke and intracerebral hemorrhage can present with features of hearing impairment, which may stem from peripheral hearing loss or central auditory processing deficits [4]. Auditory abnormalities may correlate with the site of lesion along the auditory pathway [5]. Patients with a stroke affecting their central auditory nervous system (CANS) report difficulties with sound perception, recognition, and localization extending beyond mere audibility [6]. Furthermore, several case studies have reported specific constellations of auditory symptoms associated with different areas affected by the stroke [7].

The hearing process begins with sound transduction, amplification, and the encoding of its frequency, timing and, amplitude features within the ear. This information is accurately transmitted in the auditory nerve, followed by binaural integration (important for sound localization and listening in noise) and early groupings of sounds in the brainstem which lays the foundation for ‘auditory cognitive’ processes related to auditory object formation, such as voices and speech streams, through auditory scene analysis. Incoming sounds are then matched as auditory objects to stored sound templates, dependent on context and relevance (i.e., task-dependent), to achieve sound signal recognition and formulate an appropriate behavioral response. This latter part of the process heavily relies on higher auditory cortical areas subserving language and cognitive processes [8]. Patients with Wernicke’s area stroke are reported to have auditory processing deficits [9]. Stroke can disrupt the connections in the auditory pathway, resulting in hearing difficulties ranging from impaired sound detection to altered auditory perception. When left untreated, auditory impairment can have a detrimental impact on patient communication and post-stroke rehabilitation, potentially leading to poor recovery outcomes and social isolation. There is still a scarcity of information in literature beyond case studies relating to auditory processing deficits in stroke patients [4–7, 10, 11]. It is still unclear how auditory processing deficits correspond to specific cortical and subcortical auditory brain lesions. A deeper understanding of how stroke affects these early sound processing mechanisms can provide valuable insights into stroke recovery and rehabilitation options for addressing complex language and other deficits [12]. Ultimately, this can improve the quality of life for affected patients.

The aim of this study is to determine if brain lesions (site and extent) are associated with the presence of non-speech psychoacoustic auditory processing deficits. We achieve this by comparing patients presenting with subacute stroke of the extended CANS (auditory responsive areas) with those whose CANS remains unaffected by stroke. Specifically, we investigate whether the involvement of the primary auditory cortex is associated with abnormal results in auditory processing tests. Additionally, we examine if the results of auditory processing tests correlate with the extent of CANS involvement and if there are differences in test outcomes between the group with CANS-affected stroke and the group without, while also adjusting for audiometry and cognitive confounding factors.

Our secondary aim is to explore the relationships between auditory test results, a patient-reported hearing questionnaire, and stroke severity.

## Methods

### Study design and participants

This prospective study was conducted at the Neuro-otology Department of the National Hospital for Neurology and Neurosurgery in Queen Square. The study received approval from the London Queen Square Ethics Committee (Project Identification number 11/0469 and REC ref 11/LO/1675), and written informed consent was obtained from all participants.

We recruited forty consecutive stroke patients during the subacute stage of stroke, specifically over 3 and up to 12 months after the stroke. The inclusion criteria were: a. adults aged between 18 and 80 years old b. clinical history of stroke due to ischemia or intracerebral hemorrhage, confirmed by magnetic resonance imaging (MRI) of the brain c. Pure Tone Audiogram (PTA) average (from 500 to 8000 Hz at octave levels) equal to or better than 40 dB HL in at least one ear, as hearing loss can impact some auditory processing test results [13]. Exclusion criteria were severe aphasia (defined by a cut-off score of 93.8 on the complete Western Aphasia Battery test [14], or more than mild cognitive impairment in the Montreal Cognitive Assessment (MoCA) [15], psychiatric or other neurological disorders (excluding stroke), or severe concurrent medical illnesses.

Patient were classified into those with auditory brain involvement (CANS +) and those without (CANS-) as per the extended CANS definition provided below.

### Brain magnetic response imaging (MRI)

All participants had a brain MRI performed on a 1.5 T GE Signa scanner (General Electric, Milwaukee, WI) 48 h after the stroke. The acquisition techniques included T1-weighted 3-dimensional fast low-angles shot images for volumetric and morphometric analyses. The scan acquisition parameters were as follows: repetition time = 15 ms; echo time = 5.4 ms; flip angle = 15; inversion time = 650 ms. All scans were visually inspected initially by CH in order to identify structural brain abnormalities and subsequently by RL using a checklist approach including the deep and cortical components of the auditory pathway. In patients with more than one infarct on MRI, the dominant lesion was scored separate to the non-dominant ones.

### Extended central auditory nervous system definition

The central auditory pathway was defined as consisting of the following auditory responsive structures and their connections 16: *Deep structures*: pons, medulla, thalamus-medial geniculate body. *Cortical*: Heschl’s gyrus, anterior temporal pole, superior temporal gyrus, planum temporale, supramarginal gyrus, angular gyrus, inferior parietal lobe, inferior frontal lobe and insula. *Interhemispheric connections*: corpus callosum (posterior part), anterior commissure. An independent neuro-radiologist (RL) assessed the presence or absence of involvement of each of the auditory structures by the stroke.

### CANS stroke severity index

In order to evaluate the impact of lesion load on auditory processing, a CANS stroke severity index (CANS SSI) was calculated by allocating one point for each auditory responsive area affected (as per the extended CANS definition provided above) on each side by the stroke with a score ranging from 0 (no CANS involvement) to 24. This approach was based on the methodology for our previously described infratentorial superficial siderosis imaging rating scale [17].

In addition, we graded microvascular ischemia and white matter hyperintensities (WMH) using the Fazekas scale [18]. The scale divides WMH into periventricular (PWMH) and deep (DWMH), and helps quantify small vessel disease (SVD). Each region is given a grade depending on the size and confluence of region (Table 1) and the total grade = PVWMH Grade + DWMH Grade (range 0–6) (Table 2).

**Table 1:** Fazekas Scale for Microvascular Ischemia^16^

Grade	Periventricular White Matter Hyperintensities (PVWMH)	Deep White Matter Hyperintensities (DWMH)
0	Absent	Absent
1	Cap	Punctate foci
2	Smooth halo	Early-confluent
3	Irregular and extending into the subcortical white matter	Confluent

**Table 2:** Central Auditory Nervous System Stroke Severity Index (CANS SSI)

Auditory Cortex Lesion	Score
Heschl’s gyrus	1
Anterior temporal pole	1
Superior temporal gyrus	1
Planum temporale	1
Supramarginal gyrus	1
Angular gyrus	1
Inferior parietal lobe	1
Inferior frontal lobe	1
Insula	1
Deep Auditory Brain Lesion	
Pons	1
Medulla	1
Thalamus (medial geniculate body)	1

Maximum score = 24 (sum of anatomical lesions involved; 12 for each cerebral hemisphere)

### Cognitive and audiological tests

The Montreal Cognitive Assessment (MoCA) [15] includes sections on visuospatial/executive function, naming, attention, language, abstraction, memory and orientation to time and place. A qualified neuropsychologist or a stroke specialist nurse (blind to the study) administered the MoCA.

### Standard audiometry

Standard Pure Tone Audiometry (PTA) was carried out using a GSI 61 audiometer with TDH-39 headphones (Grason-Stadler, Guymark UK Limited, West Midlands, UK). Air-conduction thresholds were measured for each ear at 0.25, 0.5, 1, 2, 4, 6, and 8 kHz following the procedure recommended by the British Society of Audiology [BSA] (2011). Results were averaged in each ear across 0.5, 1, 2, 4, and 8 kHz frequencies for the ‘PTA average’. Normal hearing thresholds were considered as 20 dB HL across the above frequency range (recommended by the BSA [2011]).

### Auditory processing assessments

Choice of tests was based on a simple hierarchical non-verbal sound processing model with increasingly complex sound representation from the periphery to the cortex, and increasing integration with other cognitive processes [19]. This model informed the creation of the Queen Square Tests of Auditory Cognition (QSTAC); see Figure S1. The main processing stages were conceptualized as the early perceptual, apperceptive and semantic levels.

The Perceptual Property Processing (PPP) [19] test assesses predominantly the cortical analysis of perceptual spectral properties. Spectral shape is a key determinant of auditory object representations (e.g., voice and instrument timbre) and supported by brain regions responsible for early perceptual coding. The patient must make a judgment of same or different for each of thirty-two sound pairs: pairs of identical sounds and pairs of different spectral shape sounds. Sounds in each pair were presented sequentially with an inter-stimulus interval of 1 s.

The Apperceptive Processing (APP) [19] test uses spectral inversion (SI) which flips or exchanges the energy present between higher and lower frequencies in a broadband sound about a user-specified frequency value to create a frequency structure that is ‘impossible’ in a natural sound. For this test, the 40 sounds (20 non-SI, 20 SI) were presented individually and for each sound, the participant was asked: ‘Is it a real thing or not a real thing?’.

The Semantic Processing (SP) [19] test examines the association of stored knowledge, or semantic memory, with perceptual (apperceptive) object representations. Thirty-two individual sounds from a range of human and animal sounds and environmental sounds were paired such that the individual sounds in a pair had dissimilar acoustic characteristics, to reduce the availability of perceptual matching cues. All 32 sounds appeared once in the ‘same’ condition (sounds produced by the same source, such as horse neighing, horse galloping) and once in the ‘different’ condition (sounds produced by different sources, such as horse neighing, human coughing). Detailed information on PP, AP and SP tests is described in Goll et al.’s study [19].

The Gaps in Noise (GIN) was also conducted [10, 20]. This test measures temporal resolution, a process that can be affected by pathology at all levels of the auditory pathway [21] by estimating the smallest just detectable perceived gap [10]. The GIN test is composed of a series of 6-s segments of broadband noise that contain 0–3 silent intervals or gaps that vary in duration between 2 and 20 ms. The GIN test compact disk was played on a Sony CD Player and passed through a GSI 61 diagnostic audiometer to TDH-39 matched earphones. The stimuli were presented at 50 dB sensation level (SL) to each ear independently. The threshold was defined as the shortest gap duration for which there were at least 50% correct identifications.

### Questionnaires

The (modified) Amsterdam Inventory for Auditory Disability and Handicap (AIAD) [22] consists of 28 items covering 5 domains (subscales) of everyday hearing ability: intelligibility of speech in noise; intelligibility of speech in quiet; auditory localization; recognition of sound; detection of sound. The response range consists of ‘almost always’ (0 point), ‘frequently’ (1 point), ‘occasionally’ (2 points), and ‘almost never’ (3 points), with a higher score denoting higher disability. A subscale score is calculated for each subscale as the sum of scores for questions answered. This has been previously used to assess auditory disability and handicap in adult patients with stroke of the CANS [6].

## Statistical analysis

We summarized continuous variables using means and standard deviations or medians and Interquartile ranges. For categorical variables, we present numbers per category (*n*) and percentage (%).

We used: a. Non-parametric Mann–Whitney test to evaluate differences in median inventory scores between case and control subjects (subjects with versus without CANS involvement, respectively); b. Fisher's Exact test to study the association between two categorical variables; c. Pearson’s partial correlation (*r*_partial_) to study the correlation between continuous test measures including auditory processing test scores, AIAD and semantic processing before and after controlling for potential confounders, including PTA, age, or both. Pearson’s partial correlation (bivariate Pearson’s partial correlation) is used to study the linear association between two continuous variables after adjusting for other continuous covariates, and measures the strength and direction of this relationship [23–26]; d. Biserial correlation (*r*_b_) to study the correlation between the dichotomous variable, presence or absence of auditory processing deficits, and the continuous variable, AIAD test score. Biserial correlation is a special case of Pearson’s correlation and is used to study the correlation when one of the variables is dichotomous with underlying continuous distribution and the other is continuous [23, 25, 26]; e. Independent samples t-test to compare the AIAD mean in patients with normal auditory processing test results. A *p*-value < 0.05 was considered statistically significant. The data analysis was performed using SPSS 26.0 for Windows (SPSS, Chicago, IL, USA).

## Results

We recruited forty consecutive patients with stroke (30 males, 10 females; age 24–78 years, mean 58.72 years) who met the study’s inclusion criteria from the stroke unit at National Hospital for Neurology and Neurosurgery (NHNN) and the hyper-acute stroke unit (HASU) at University College London Hospitals (UCLH). These patients were assessed at the Department of Neuro-otology, NHNN Queen Square, within three to twelve months after the onset of their stroke (33 ischemic, 7 hemorrhagic; 33 cortical, 7 subcortical). The age range of the participants within the auditory brain stroke group was 24 to 77 years, with a mean age of 57.63 (SD 16.134), and 44 to 78 years in the non-auditory stroke group, with a mean age of 61 (SD 11.460). Of the 40 stroke patients, 20 patients had right hemisphere lesions, 18 had left hemisphere lesions, and 2 had bilateral stroke lesions (hemorrhagic, one with auditory and another with non-auditory stroke). 27 had auditory brain areas affected by the stroke: 20 had cortical involvement (including 3 cases with non-dominant stroke), and 7 had subcortical involvement (including 2 with non-dominant stroke). There was no significant difference in lesion side between those with (right: 11; left: 15; bilateral: 1) and without auditory involvement (right: 9; left: 3; bilateral: 1) (Fisher’s exact test, *p* = 0.126). Our study included 28 subjects with unilateral or bilateral abnormal GIN (70%; 17 with CANS stroke), 19 with abnormal PPP (47.5%; 16 with CANS stroke), 17 with abnormal APP (42.5%; 10 with CANS stroke), and 5 with abnormal SP (12.5%; 2 with CANS stroke). Table 3 summarizes the distribution of different measures between the auditory and non-auditory stroke groups.

**Table 3:** Descriptive Statistics of Different Measures Between Auditory (CANS +) and Non-auditory (CANS-) Stroke Groups

	Mean (SD); Median		Percentage of Abnormal	
	CANS +	CANS –	CANS +	CANS –
Age	57.63 (16.134); 63	61(11.460); 64	–	–
R PTA average (dBHL)	22.2 (12.31); 21.66	24.0 (11.38); 23.0	14/27 (52%)	9/13 (69%)
L PTA average (dBHL)	22.28 (13.32); 20.83	23.9 (11.7); 26.6	14/27 (52%)	8/13 (61.5%)
R GIN threshold (ms)	7.64 (1.84); 8	8.0 (2.16); 8	14/27 (52%)	9/13 (69%)
L GIN threshold (ms)	7.75 (1.77); 8	8.23 (2.45); 8	16/27 (59%)	9/13 (69%)
Perceptual property processing (PPP; total score)	28.55 (3.26); 29	30.69 (1.37); 31	16/27 (59%)	3/13 (23%)
Apperceptive (APP; total score)	37.07 (3.04); 27	35.15 (5.32); 13	10/27 (37%)	7/13 (54%)
Semantic (SP; total score)	30.96 (1.55); 27	29.53 (3.71); 13	2/27 (7%)	3/13 (24%)
MoCA	24.75 (3); 25	26.4 (2.1); 26	10/20 (50%)	4/12 (33%)
Fazekas total score	2.75 (1.99); 2	2.83 (1.85); 2.83	Score 1: 10/20 (50%) Score 2: 10/20 (50%)	Score 1: 5/12 (42%) Score 2: 7/12 (58%)

*CANS* central auditory nervous system; *dBHL* decibel hearing level; *GIN* gaps in noise; *MoCA* Montreal Cognitive Assessment; *PTA* pure tone audiometry; *SD* standard deviation; *R* right; *L* left

Mann–Whitney U test was conducted to assess potential differences in the average worse ear PTA, age and individual scores on the auditory processing test between the auditory and non-auditory stroke groups. The distributions of average worse ear PTA, age and auditory processing test battery scores were found to be similar. The median values for average worse ear PTA, age and individual auditory processing test scores did not show statistically significant differences between auditory and non-auditory stroke groups.

Similarly, the median Fazekas score for auditory stroke and non-auditory stroke did not exhibit a statistically significant difference based on Mann–Whitney (*p* = 0.716).

### Heschl’s gyrus involvement and early perceptual (PPP) scores

Thirty-four patients had isolated auditory responsive cortical area involvement only. Within the cortical auditory stroke group, when comparing patients with Heschl’s gyrus involvement to those without, it was found that patients with Heschl’s gyrus involvement had worse early perceptual (PPP) scores (*p* = 0.048). The distribution of other individual auditory processing disorder test scores was similar in both groups (*p* > 0.05).

#### Characteristics of patients with normal auditory processing test results

Nine patients exhibited normal results in GIN, PPP, APP, and SP tests, while 15 patients had abnormal GIN results, even though their PPP, APP and SP results fell within the normal range (see APPENDIX: 1. Characteristics of Patients with Auditory and Non-auditory Brain Lesions). The nine patients who showed no evidence of abnormalities in their auditory processing test results did not significantly differ from the remaining group in terms of age (*p* = 0.2), WMHt score (*p* = 0.79), auditory cortical involvement (*p* = 0.28), or auditory involvement (*p* = 1). However, they reported significantly less auditory impairments on the AIAD (mean difference 11.5, *p* < 0.005). None of these patients had Heschl’s gyrus involvement, and only one had superior temporal gyrus involvement.

### CANS stroke severity index and auditory processing test scores

**Fig. 1:**
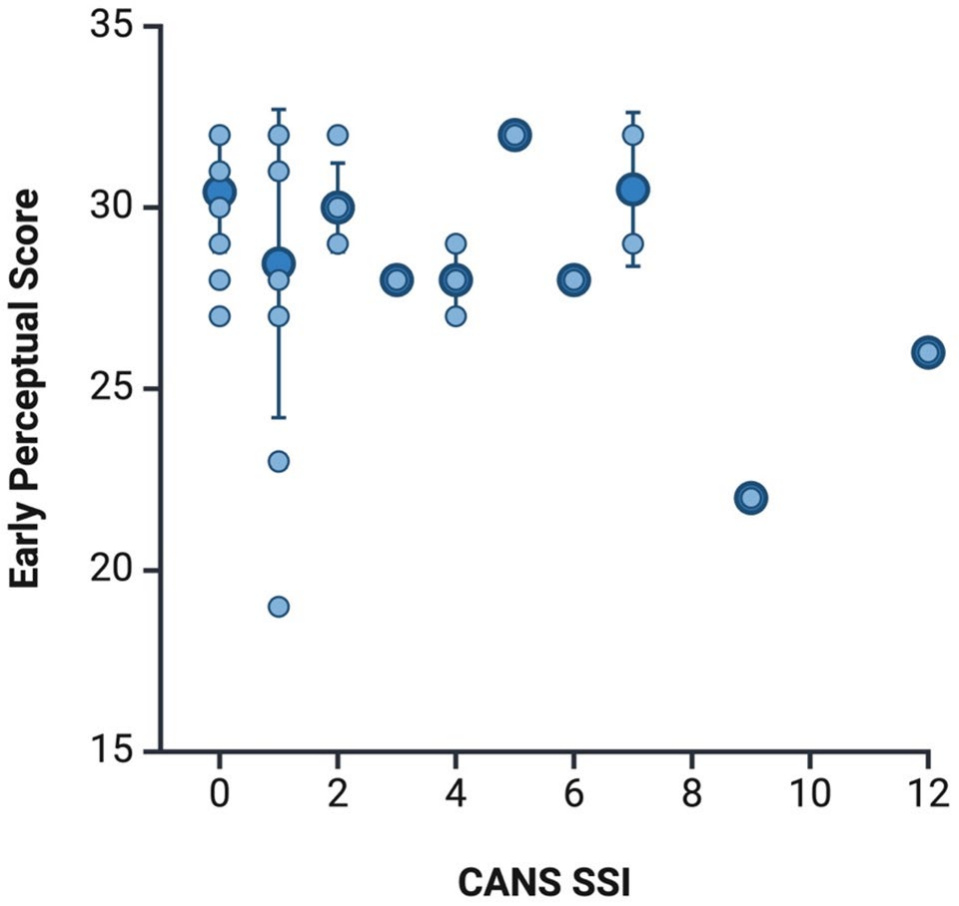
Simple Scatter of Early Perceptual Score by CANS Stroke Severity Index

Pearson’s partial correlation was conducted to assess the relationship between CANS SSI and auditory processing test results. The analysis revealed a negative correlation between SSI and the early perceptual (PPP) score, indicating that as CANS SSI increased, the early perceptual score decreased (*r*_partial_ = – 0.313, *p* = 0.049) (see Fig. 1). The strength of this correlation was more when age and PTA in the worse ear were controlled for, *r*_partial_ = – 0.412, but still statistically significant, *p* = 0.010. However, there was no statistically significant relationship between SSI and other auditory processing test scores (Tables 4, 5).

**Table 4:** Pearson’s Partial Correlation between test scores results and the patient self-reported auditory difficulties on the AIAD and MoCA

	PTAw	GINw	AIAD	MoCA	CANS SSI	PPP	APP	Semantic	Age
PTAw									
Correlation	1.00	0.403	0.340	– 0.468	– 180	– 0.249	– 0.338	– 0.330	0.688
*P*-value	–	**0.027**	0.**032**	**0.009**	0.340	0.185	0.068	00.75	**0.000027**
GINw									
Correlation	0.403	–	0.234	– 0.247	– 0.018	– 0.458	– 0.242	– 0.084	0.250
*P*-value	**0.027**	–	0.214	0.189	0.923	**0.011**	0.198	0.659	0.183
AIAD									
Correlation	0.340	0.234	1.000	– 0.337	0.155	– 0.112	– 0.238	– 0.219	0.103
*P*-value	**0.032**	0.214	–	0.069	0.415	0.556	0.205	0.245	0.589
MoCA									
Correlation	– 0.468	– 0.247	0.337	1.000	– 0.418	0.414	0.328	0.328	– 0.257
*P*-value	**0.009**	0.189	0.069	–	**0.022**	**0.023**	0.076	0.077	0.171
CANS SSI									
Correlation	– 0.169	– 0.018	0.155	– .418	1.000	– 0.477	– 0.101	– 0.171	– 0.492
*P*-value	0.372	0.923	0.415	**.022**	–	**0.008**	0.594	0.366	**0.006**

The bold numbers indicate the statistically significant values

*PTAw* pure tone audiometry in worse ear; *AIAD* (Modified) Amsterdam Inventory for Auditory Disability and Handicap; *CANS SSI* central auditory nervous system stroke severity index; *MoCA* Montreal Cognitive Assessment; *EP* early perceptual score; *APP* apperceptive processing; Semantic, semantic processing; *GINw* gaps-in-noise test in the worse ear

**Table 5:** Pearson’s Partial Correlation between test scores results and the patient self-reported auditory difficulties on the AIAD and MoCA after controlling for age and PTA in the worse ear

	GINw	AIAD	MoCA	CANS SSI	PPP	APP	Semantic
GINw							
Correlation	1.000	0.114	– 0.068	0.041	– 0.404	– 0.126	0.050
*P*-value	–	0.562	0.732	0.837	**0.033**	0.522	0.800
AIAD							
Correlation	0.114	1.000	– 0.209	0.165	0.000	– 0.161	– 0.162
*P*-value	0.562	–	0.286	0.401	0.999	0.413	0.410
MoCA							
Correlation	– 0.068	– 0.209	1.000	– 0.593	0.336	0.214	0.231
*P*-value	0.732	0.286	–	**0.001**	0.081	0.274	0.238
CANS SSI							
Correlation	0.041	0.165	– 0.593	1.000	– 0.518	– 0.232	0.048
*P*-value	0.837	0.401	**0.001**	–	**0.005**	0.234	0.810

*PTAw* pure tone audiometry in worse ear; *AIAD* (Modified) Amsterdam Inventory for Auditory Disability and Handicap; *CANS SSI* Central auditory nervous system stroke severity index; *MoCA* Montreal Cognitive Assessment; *PPP* perceptual property processing score; *APP* apperceptive processing; Semantic, semantic processing; *GINw* gaps-in-noise test in the worse ear

The bold numbers indicate the statistically significant values

### Semantic processing deficits

No patient had isolated semantic processing deficits. Five patients who had semantic deficits (2 with CANS stroke) also had:

- apperceptive deficits with bilaterally abnormal GIN: in two cases (bilateral caudate heads, lentiform and corona radiata; right cingulate gyrus and non-dominant (ND) left lingual gyrus of occipital lobe), and
- early perceptual as well as apperceptive deficits in three cases
  o with bilaterally abnormal GIN in two (right cuneus and lingual gyrus of occipital lobe, right parahippocampal gyrus, right fusiform gyrus, right splenium of corpus callosum, ventral lateral right thalamus; left supramarginal and angular gyrus)
  o and GIN within normal limits in one – left pontine tegmentum.

### Apperceptive deficits

Six patients exhibited apperceptive deficits without early perceptual (PPP) deficits (2 with CANS stroke). Those included:

1. A 77-year-old male with an ischemic stroke in the deep auditory area involving the left pons. He had moderate peripheral hearing loss and bilateral abnormal GIN.
2. A 57-year-old male with a left ischemic stroke in the auditory cortex with normal peripheral hearing thresholds and abnormal left GIN. Stroke lesions involved Heschl's, supramarginal and long insular gyri.
3. A 78-year-old male with a non-auditory ischemic stroke in the non-dominant lobe. This involved the left cingulate gyrus and left lingual gyrus of occipital lobe. He had mild peripheral hearing loss and abnormal GIN bilaterally.
4. A 72-year-old female with bilateral hemorrhagic stroke involving the caudate heads, lentiform and corona radiata bilaterally and sparing the CANS. She had mild peripheral hearing loss and bilateral abnormal GIN.
5. A 68-year-old male with a right ischemic stroke with anterior striatal involvement (caudate and putamen). The non-auditory central nervous system was not affected. He had mild peripheral hearing loss and normal GIN.
6. A 44-year-old male with a right ischemic stroke involving the CANS including the Heschl's gyrus, superior temporal gyrus, planum temporale, supramarginal gyrus, insula, frontal—superior, middle and inferior, orbital gyri, gyrus rectus. Other affected areas included the posterior caudate, putamen and corona radiate. He had normal peripheral hearing and abnormal bilateral GIN.

In patients with isolated PPP deficits, none (0/8) had caudate involvement. In patients with APP (+/- SP) but without PPP deficits, four (4/5) had caudate involvement. Fisher's exact test was conducted between caudate involvement and APP deficits. There was a statistically significant association between caudate involvement and APP deficits (*p* = .007).

### Correlations between AIAD, auditory test measures and CANS SSI

Pearson’s partial correlation revealed a negligible correlation between the average PTA in the worse ear and the AIAD (*r* = 0.340, *p* = 0.032). This correlation was stronger after controlling for age (*r* = 0.423, *p* = 0.007). Additionally, a biserial correlation demonstrated a statistically significant, low positive correlation between the presence or absence of auditory processing deficits (AP deficits) and AIAD scores in stroke patients (*r*_b_ = 0.536, *p* = 0.013).

Pearson's partial correlation was run to assess the relationship between each of the test results, the patient self-reported auditory difficulties on the AIAD and the MoCA.

The average PTA in the worse ear exhibited a low negative correlation with MoCA and a moderate positive correlation with age (Table 4). A bivariate Pearson’s correlation showed low negative correlations between CANS SSI, MoCA and early perceptual score (Table 4). There was no significant correlation between early perceptual scores and MoCA when controlling for age and average PTA in the worse ear (Table 5). The linear relationship between MoCA and stroke SSI shifted from low (Table 4) to moderate when age and average PTA in the worse ear were controlled for (Tables 4, 5). Average PTA in the worse ear displayed a weak positive correlation with worse ear GIN (Table 4), but this did not reach statistical significance when controlling for age (*p* = 0.064).

## Discussion

Previous studies have reported significant functional difficulties in everyday listening and related tests that are associated with central auditory nervous system involvement in patients with stroke [7, 8]. However, to date, no study has examined the impact of both the location and severity of stroke on early sound processing tests, while considering baseline audiometry, cognition, language, and patient-reported symptom questionnaires. This information is essential for distinguishing the relative effects of central auditory dysfunction on psychoacoustic tests and patient symptoms from higher-level cognitive, language, and other deficits, as well as ‘peripheral’ hearing loss. Such insights will enhance our understanding of the mechanisms contributing to different stroke-related behavioral effects and aid in the development of rehabilitation strategies for communication deficits [12].

Although we did not find statistically significant differences in AP deficit between the CANS + and CANS- groups on initial analysis, this is probably because the simple division between both groups does not capture the complexity of lesion severity and the extent of central auditory involvement. Small vessel disease [27] and other functional abnormalities that may not be detectable with standard imaging techniques could obscure the relationship between lesion locations and auditory processing outcomes. These changes could affect subcortical and white matter pathways important for auditory processing. Moreover, lesions in the posterior circulation territory, such as those in the brainstem or thalamus, even if not directly involving auditory cortex regions, may impact adjacent areas critical for transmitting and processing auditory signals, without visible evidence on MRI. These areas often share vascular networks with auditory pathways, and disruptions in these regions may indirectly impair auditory function. These microvascular changes or functional disruptions might contribute to auditory deficits that are not fully reflected by either imaging or the tests employed in this study. Upon further inspection of the data, we observed that when we accounted for CANS lesion severity, using the CANS SSI, patients with more severe involvement tended to demonstrate worse performance on early perceptual processing tasks (PPP). This suggests that the extent of CANS involvement, rather than merely the presence or absence of CANS lesions, may play a more crucial role in influencing auditory outcomes.

Our results demonstrated that patients with involvement of the primary auditory cortex (Heschl's gyrus) had worse early perceptual scores compared to those without such involvement, even when considering similar cognitive, language, and audiometric findings in both groups. A higher CANS SSI, indicating a greater load of auditory lesions, correlated with worse early perceptual scores. The correlation strengthened when we controlled for age and the average PTA in the worse ear. In contrast, we found no correlation between early perceptual scores and MoCA when age and average PTA in the worse ear were controlled for. These findings align with the proposed hierarchical non-verbal sound processing model by Johnson et al. [16] in which the analysis of detailed spectro-temporal structure (early perceptual processing) is critically dependent on auditory cortices but also strongly affected by subcortical pathways. Our findings emphasize the significance of the primary auditory cortex and lower-level CANS in early perceptual sound processing, particularly after accounting for the effects of peripheral hearing loss or cognitive factors. Furthermore, the observation that the MoCA scores were not associated with early perceptual impairment, as assessed by the PPP, after controlling for age and audiometric thresholds, supports the notion that this specific auditory test reflects perceptual irregularities rather than ‘top-down’ processes, as previously demonstrated [28]. Given that the stroke territory of those with Heschl’s gyrus lesions is similar in this retrospective data, we could interpret the statistical analysis results on comparing the AP deficits within cortical CANS stroke in Heschl’s gyrus lesions to cortical CANS stroke sparing the Heschl’s gyrus as discussed above. Four out of the five patients with Heschl's gyrus involvement also had temporal gyrus involvement and those had an abnormal PPP. The fifth patient had Heschl’s gyrus stroke without superior temporal gyrus involvement and the PPP was normal. All of the five patients had supramarginal gyrus and insula involvement. It was difficult to study other anatomical lesions and their association with specific AP deficits because the stroke territory for the other lesions was more variable in our sample, and some lesions were found in only one or two patients (APPENDIX: 1. Characteristics of Patients with Auditory Brain Lesions).

Interestingly, abnormal GIN results were slightly more common in the CANS- group compared to the CANS + , a test that reflects temporal resolution but may also be affected by attention/executive function deficits. Surprisingly, there was no correlation observed between CANS SSI and the GIN score. This lack of correlation may be attributed to the GIN’s sensitivity to lesions at various levels of the auditory pathway [21]. Even a lower-level CANS lesion can significantly impair time-based (temporal) information encoding and GIN performance without cumulative effects from higher lesions. A related point is that, unlike for verbal auditory stimuli which are highly lateralized, non-verbal auditory processing is largely bilateral. This means that even in cases where an individual patient has a unilateral lesion affecting a brain region supporting GIN processing, the homologous region in the other hemisphere may compensate. Furthermore, the GIN revealed a weak positive correlation with audiometric thresholds. However, this correlation lost significance when age was taken into account. This loss of significance would be expected in view of the strong degrading effect of aging on neurotransmitter pathways necessary for temporal processing [29]. Additionally, GIN may be influenced by factors such as small vessel disease, which can cause microvascular changes not visible on standard imaging, as previously discussed. This can particularly affect temporal processing, as captured by the GIN test. Of the eight CANS- patients, four with posterior circulation strokes (Patients 30, 34, 36, and 39) had abnormal GIN results, suggesting that lesions in the posterior circulation territory, while not directly located in primary auditory areas, still affect auditory temporal processing. This may occur through shared vascular networks or disruptions in adjacent brainstem, thalamic, or subcortical pathways that support auditory functions. Also, these auditory processing abnormalities may not be fully reflected on standard MRI imaging as microvascular changes or subtle disruptions in white matter pathways may go undetected. For patients with lesions in non-auditory areas (APPENDIX: 2. Explanation of abnormal gaps-in-noise (GIN) test findings for patients with lesions in non-auditory areas), such as the corona radiata, lentiform nucleus, or thalamus (e.g., Patients 28 and 35), their abnormal GIN results are likely due to disruption of subcortical sensory integration pathways or white matter tracts that indirectly support auditory functions. Although these lesions are not in primary auditory areas, they are in proximal regions that interact with or support broader networks involved in auditory processing. For example, Patient 28's lesion in the posterior corona radiata likely impacts white matter tracts that are crucial for transmitting signals between the auditory cortex and other parts of the brain, causing impaired temporal auditory processing. In some cases, such as with Patient 34 (lesions in visual areas), the exact cause of the GIN abnormality remains uncertain, as these regions do not typically contribute to auditory processing.

Interestingly, we found no correlation between CANS SSI and APP and SP scores. These tests are more likely to reflect the function of cognitive brain areas [19]. This finding is consistent with the observation that involvement of the caudate nucleus, a key region for memory, [30] was more common in those with apperceptual processing deficits compared to those without. This highlights the role of the caudate in temporal structure analysis and in extraction of a categorization ‘rule’ from novel stimuli, which is not specifically auditory [31]. We note that the APP and SP tests were even more often impaired in the CANS- group than the CANS + group, possibly reflecting the more widespread generalized cognitive impairment in that group: both of these tests have executive components. It is unlikely, however, that this effect is attributable to reduced auditory working memory in the CANS- group as the PPP and SP tests have fairly similar working memory demands, whereas the APP test is not as loaded on working memory.

Assessing the auditory processing pathway is particularly challenging in the stroke population, who may have concurrent peripheral hearing loss and central auditory disorders [Gates et al. 32]. The former may result from pre-existing presbycusis, stroke- or vascular-related peripheral hearing loss, or both [33] along with impairments of higher-order functions due to aging. Humes et al. [27] suggested that central presbycusis is multifactorial and results from peripheral and central ‘age and/or disease-related changes’. To account for these considerations, we adjusted for age and PTA as potential confounding factors and measured small vessel disease in our study group as an additional measure of age and/or disease-related changes [34]. Since there was no statistical difference in either audiometric thresholds, cognitive factors (MoCA), or SVD severity between the auditory and non-auditory stroke groups, these factors are not likely to be the primary driver of our results.

Reassuringly, audiometric thresholds did not correlate with PPP, APP, and SP tests. This suggests that these tests are ‘suprathreshold’ tasks that are less reliant on hearing threshold problems. They can be clinically used to reliably assess patients, even those with mild hearing loss. Similar to previous reports [35], MoCA scores were found to correlate with audiometric thresholds. This will need to be considered when assessing cognition in these patients and will affect choice of acoustic environment, consideration of amplification or the use of a modified MoCA that is less dependent on hearing function [33].

Patient-reported auditory disability, as assessed by the AIAD, showed a weak correlation with the presence of auditory processing test deficits. However, it was not predicted by cognitive function (MoCA). Our results indicate that assessment of hearing acuity, auditory processing and cognitive function tap into overlapping yet distinct behaviors and communication needs. These findings emphasize the importance of a holistic approach in rehabilitation planning to address the overall communication and wellbeing of stroke patients. Therefore, our results have implications for the clinical management of stroke patients. While conducting an exhaustive, detailed audiological assessment for every single stroke patient may be impractical, patients with high levels of auditory disability in the presence of CANS involvement should receive additional investigation and specialized care.

Our study has some limitations that are worth noting. These include a relatively small number of patients and a retrospective design, which did not allow for a volumetric brain approach. The use of a test battery approach, as opposed to a symptom-driven and custom-made test approach, could have potentially led to an under-identification of more specific deficits associated with different lesions. Furthermore, we excluded patients with severe aphasia to ensure that those unable to understand the information letter, consent, and test instructions were excluded. However, this criterion is likely to bias the sample toward more right hemisphere lesions compared to left, and we may have missed some findings of interest. In addition, the CANS + group included patients with a variety of different lesions within the CANS. The differences between this group and the CANS- group might have been obscured for this reason. Considering these limitations, the strongest signal is the abnormal PPP performance in the group with CANS involvement compared to the group without CANS involvement as the PPP test is the most modality-specific of the auditory cognition tests we conducted. APP and SP tests are not specifically auditory—both entail top-down processing from executive and multimodal semantic mechanisms. This is reflected in the lack of clear discrimination of these tests for patients with and without CANS involvement. In contrast, the GiN correlates with both PTA and PPP, reflecting both peripheral and central hearing effects. None of the ‘central’ hearing tests we conducted is an effective predictor of daily life hearing symptoms on the AIAD in those with non-aphasic stroke. This suggests that other auditory processes not captured by the tests used may be more important to daily life hearing function in this stroke population which involve the brain's ability to segregate and process multiple sound sources in a complex environment i.e., aspects of auditory scene analysis. Larger studies with an expanded test battery and more systematic imaging analysis with a volumetric approach would be helpful in future to gain a better understanding of the auditory processing deficits in stroke patients. Also, future studies with a design including a stroke population enriched for infarcts involving the CANS, such as Heschl’s gyrus, temporoparietal lobe and insula, would be helpful to further understand how the auditory brain is affected by cerebrovascular disease, and study the association between different types of strokes and hearing changes. Furthermore, advanced imaging techniques, such as functional MRI or perfusion MRI, could also provide a more comprehensive understanding of the impact of small vessel disease on auditory processing deficits in stroke patients. Consequently, further research is necessary to replicate and extend these findings.

## Conclusion

To the best of our knowledge, this is the first study to demonstrate the correlation between stroke severity in terms of higher number of lesions involving auditory areas in patients with subacute stroke and worse early perceptual (PPP) and AIAD scores. Notably, our findings suggest a potential association between Heschl's gyrus involvement and a decline in early perceptual scores. We believe our study contributes to our understanding of stroke affecting the CANS and highlights the importance of early auditory assessment and targeted interventions, offering valuable insights into stroke care and research.

## Supplementary Information

Below is the link to the electronic supplementary material.

Supplementary file1 (DOCX 31 KB)

Supplementary file2 (DOCX 461 KB)

## Data Availability

Anonymised data on excel available by emailing first author.
