# Supplementary material for: Auditory processing deficits in subacute stroke
Source: J Neurol. 2024 Dec 16;272(1):80. doi: 10.1007/s00415-024-12754-x (PMC11649831; doi:10.1007/s00415-024-12754-x)
Supplement: Supplementary file 1 — Supplementary file1 (DOCX 31 KB) [file 415_2024_12754_MOESM1_ESM.docx]

**APPENDIX**

1. **Characteristics of Patients with Auditory and Nonauditory Brain Lesions. KEYS: PPP, perceptual property processing score; APP, apperceptive processing; GIN, gaps-in-noise test.**

| **Serial Number** | **Auditory (A)/ Non-auditory (NA)** | **Side of stroke (R = Right; L = Left; B = Bilateral)** | **Ischemic (I) /Haemorrhagic (H)** | | **Dominant (D)/non-dominant (ND) stroke** | | **Cortical (C)/Deep (D)** | | **Site(s) of subacute auditory stroke lesion(s)** | | **Sites of subacute non-auditory stroke lesion(s)** | **Old stroke lesion(s)** | **Average PTA in worse ear (dB HL)** | **APD tests with abnormal results** |  |
| --- | --- | --- | --- | --- | --- | --- | --- | --- | --- | --- | --- | --- | --- | --- | --- |
| 1 | A | L | | I | | D | | C | | Heschl's, superior temporal, supramarginal and long insular gyri | None | None | 40 | GIN, PPP | |
| 2 | A | R | | I | | D | | C | | Heschl's, superior temporal gyrus, supramarginal gyrus, angular gyrus, insula | Middle and inferior temporal gyri, post-central, subcentral, precentral gyri, inferior frontal gyri, right striatocapsular (partly haemorrhagic) | None | 37.5 | PPP, APP | |
| 3 | A | L | | I | | D | | C | | Supramarginal and angular gyrus | None | None | 27.5 | GIN, PPP, APP, SP | |
| 4 | A | L | | H | | D | | D | | Pons | Left cerebellar haemorrhage | None | 55.83 | GIN, PPP | |
| 5 | A | L | | I | | D | | C | | Short insular gyri, middle frontal gyrus, pars triangularis and opercularis of inferior frontal gyrus | None | None | 17.50 | GIN, PPP, APP | |
| 6 | A | L | | I | | D | | C | | Inferior parietal lobule, temporal operculum, posterior insula | None | None | 13.33 | PPP | |
| 7 | A | L | | I | | D | | C | | Middle frontal gyrus | Left cingulate gyrus | None | 4.17 | None | |
| 8 | A | L | | I | | ND | | C | | Supramarginal gyrus | Bilateral cerebellar hemispheric, right putamen | None | 54.17 | PPP | |
| 9 | A | R | | I | | ND | | C | | Insular cortex | Right precentral gyrus, corona radiata and centrum semiovale | None | 30.00 | GIN, PPP | |
| 10 | A | L | | I | | ND | | D | | Dorsolateral medulla | Left hippocampus, parahippocampal gyrus and fusiform gyrus | None | 29.17 | GIN | |
| 11 | A | R | | I | | ND | | D | | Dorsolateral medulla | Right cerebellar hemisphere, Right dentate nucleus, Right precentral gyrus and Right posterior superior frontal gyrus | None | 22.50 | None | |
| 12 | A | R | | H | | D | | D | | Ventral lateral medulla | None | None | 18.33 | GIN, PPP, APP | |
| 13 | A | L | | I | | ND | | C | | Heschl's, Superior temporal, Planum temporale, supramarginal gyrus, insula, frontal - superior, middle and inferior, orbital gyri, gyrus rectus | Left globus pallidus, internal capsule - genu and posterior limb, left hippocampus | None | 18.33 | GIN, PPP, APP | |
| 14 | A | R | | I | | ND | | C | | Superior temporal gyrus (inferior posterior cortex), supramarginal gyrus, angular gyrus | Right superior and middle occipital gyri | None | 10.00 | GIN, PPP, APP | |
| 15 | A | R | | I | | ND | | C | | Inferior frontal gyrus, superior temporal gyrus, insula | Right putamen and caudate | None | 8.33 | None | |
| 16 | A | L | | I | | D | | C | | Insula, Heschl's, Superior posterior temporal gyrus, Supramarginal gyrus | Right corona radiata | None | 7.50 | GIN, PPP | |
| 17 | A | B | | H | | D | | D | | Central pons | None | None | 4.17 | None | |
| 18 | A | R | | I | | ND | | C | | Superior frontal gyrus | Right middle cerebellar peduncle | None | 40.00 | GIN, PPP | |
| 19 | A | L | | I | | D | | D | | Left hemipons | None | None | 45.83 | GIN, APP | |
| 20 | A | R | | I | | D | | C | | Superior frontal gyrus | Right precentral gyrus | None | 31.67 | GIN, PPP, APP | |
| 21 | A | L | | H | | D | | C | | Supramarginal gyrus, angular gyrus | Postcentral gyrus | None | 27.50 | GIN | |
| 22 | A | R | | I | | ND | | C | | Supramarginal gyrus | Right precentral gyrus (hand knob), right postcentral gyrus | None | 25.83 | None | |
| 23 | A | R | | I | | ND | | C | | Supramarginal gyrus, angular gyrus | Right precentral gyrus, post central gyrus, superior parietal lobule, right superior and middle occipital gyri | None | 28.33 | GIN, PPP | |
| 24 | A | R | | I | | ND | | C | | Middle frontal gyrus | Right precuneus, right lingual gyrus | Ischemic infarct of right head of caudate, right thalamus | 21.67 | GIN | |
| 25 | A | L | | H | | D | | C | | Supramarginal gyrus | Right cuneus of occipital lobe | None | 19.17 | None | |
| 26 | A | L | | I | | D | | C | | Heschl's, supramarginal, long insular gyri | Left body of caudate and corona radiata | None | 16.67 | GIN, APP | |
| 27 | A | L | | I | | D | | D | | Left pontine tegmentum | None | None | 12.50 | PPP, APP, SP | |
| 28 | NA | L | | I (H transformation) | | D | | - | | - | Left head of caudate, anterior limb of internal capsule, lentiform nucleus, left posterior corona radiata | None | 40 | GIN | |
| 29 | NA | R | | I | | D | | - | | - | Right pre central, post central and subcentral gyri | None | 35 | GIN | |
| 30 | NA | R | | I | | ND | | - | | - | Ventromedial thalamus | Left cerebellar hemisphere ischemic infarct | 22.5 | GIN | |
| 31 | NA | L | | I | | D | | - | | - | Left cuneus and lingual gyrus of occipital lobe | None | 10 | None | |
| 32 | NA | R | | I | | ND | | - | | - | Right ventrolateral thalamus and posterior limb internal capsule | Right corona radiata and putamen lacunar infarct | 25.8 | None | |
| 33 | NA | L | | I | | D | | - | | - | Left posterior caudate, putamen and corona radiata | None | 6.67 | None | |
| 34 | NA | R | | I | | ND | | - | | - | Cingulate gyrus | Ischemic infarct of left lingual gyrus of occipital lobe | 30.83 | GIN, APP, SP | |
| 35 | NA | R | | H | | D | | - | | - | Right lentiform nucleus | None | 27.50 | GIN, APP, PPP | |
| 36 | NA | R | | I | | D | | - | | - | Right facial colliculus and/or medial longitudinal fasciculus |  | 40.00 | GIN, APP, PPP | |
| 37 | NA | B | | H | | D | | - | | - | Bilateral caudate heads, lentiform and corona radiata | None | 36.67 | GIN, APP, SP | |
| 38 | NA | R | | I | | D | | - | | - | Right anterior striatal (caudate + putamen) | None | 26.67 | APP | |
| 39 | NA | R | | I | | ND | | - | | - | Right cuneus and lingual gyrus of occipital lobe, right parahippocampal gyrus, right fusiform gyrus, right splenium of corpus callosum, ventral lateral right thalamus | Ischemic stroke of right anterior head of caudate, anterior limb of internal capsule and anterior putamen (anterior striatocapsular) | 20 | GIN, PPP, APP, SP | |
| 40 | NA | R | | I | | D | | - | | - | Posterior caudate, putamen and corona radiata |  |  | GIN, APP | |

1. **Explanation of abnormal gaps-in-noise (GIN) test findings for patients with lesions in non-auditory areas**

| Patient | Lesion location | Explanation for abnormal GIN |
| --- | --- | --- |
| 28 | Left head of caudate, anterior limb of internal capsule, lentiform nucleus, left posterior corona radiata | Involvement of the corona radiata disrupts white matter tracts connecting auditory regions, likely impairing temporal processing. Microvascular disease may also affect subcortical auditory pathways. |
| 29 | Right precentral, postcentral, and subcentral gyri | Primarily motor and sensory areas. Possible involvement of small vessel disease or disrupted multisensory integration could impair auditory temporal processing, though the exact reason for GIN abnormality remains uncertain. |
| 30 | Ventromedial thalamus (posterior circulation stroke) | The thalamus is involved in sensory relay, including auditory information. Disruption in thalamocortical networks may indirectly affect auditory temporal processing. Also, although the lesions may not be directly within classical auditory areas, they are located in nearby regions and share vascular circulations that support auditory pathways. |
| 34 | Left cuneus and lingual gyrus (posterior circulation stroke) | Primarily visual areas. Although the lesions may not be directly within classical auditory areas, they are located in nearby regions and share vascular circulations that support auditory pathways. |
| 35 | Right lentiform nucleus | The lentiform nucleus (basal ganglia) plays a role in timing and sequencing essential for auditory processing. Disruption here may impair temporal auditory processing. Small vessel disease may also contribute. |
| 36 | Right facial colliculus (pons) (posterior circulation stroke) | Posterior circulation stroke affecting brainstem auditory pathways. Although the lesions may not be directly within classical auditory areas, they are located in nearby regions and share vascular circulations that support auditory pathways. |
| 37 | Bilateral caudate heads, lentiform nucleus, and corona radiata | Lesions in the basal ganglia and corona radiata likely disrupt subcortical sensory integration pathways, impacting temporal processing. White matter tracts and small vessel disease may further contribute to the GIN abnormality. |
| 39 | Right cuneus, lingual gyrus, parahippocampal gyrus, fusiform gyrus, splenium of corpus callosum, ventral lateral thalamus (posterior circulation stroke) | Multiple areas involved, including the thalamus, which plays a role in sensory integration. Although the lesions may not be directly within classical auditory areas, they are located in nearby regions and share vascular circulations that support auditory pathways. |
