# Supplementary material for: Auditory processing deficits in subacute stroke
Source: J Neurol. 2024 Dec 16;272(1):80. doi: 10.1007/s00415-024-12754-x (PMC11649831; doi:10.1007/s00415-024-12754-x)
Supplement: Supplementary file 2 — Supplementary file2 (DOCX 461 KB) [file 415_2024_12754_MOESM2_ESM.docx]

**Supplementary material**

**Figure S1.**

**
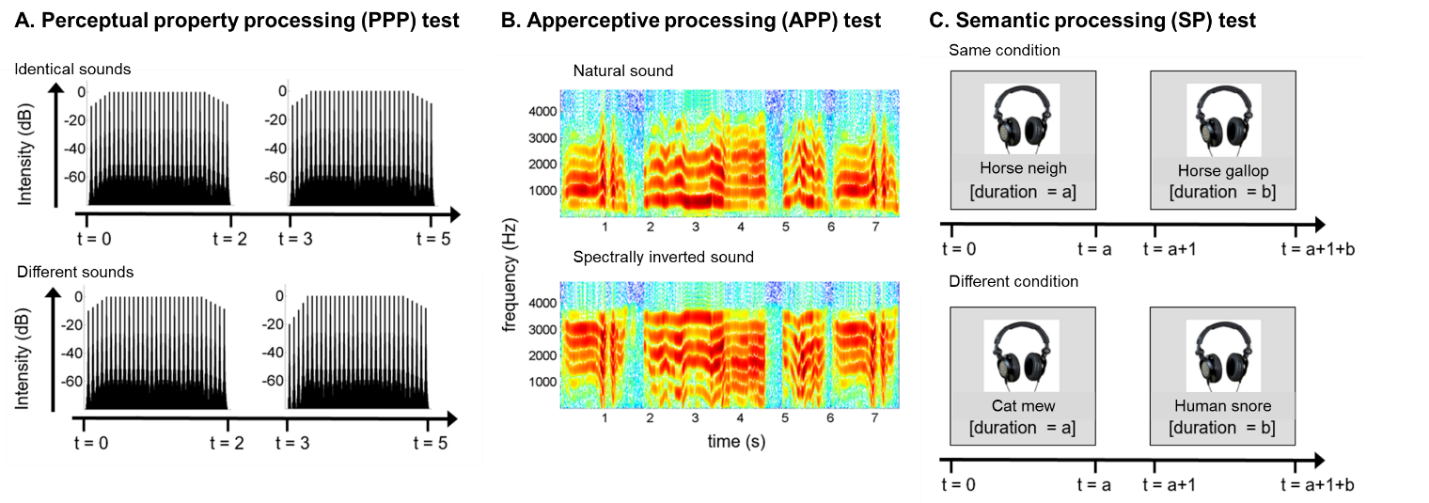
**

Schematic of experimental stimuli and presentation sequences for subtests from the Queen Square Tests of Auditory Cognition (QSTAC) battery. (A) The perceptual processing property (PPP) test assesses processing of spectral shape: the patient must judge whether sound pairs represent the ‘same’ (top panel) or ‘different’ (bottom panel) sounds. Sounds in each pair were presented sequentially with an inter-stimulus interval of 1s. (B) Apperceptive processing (APP) test: the patient must judge whether a presented sound is ‘real’ (natural; top panel) or ‘not real’ (spectrally inverted; bottom panel. Spectrally inverted stimuli were created by exchanging the energy present between higher and lower frequencies in a broadband sound to create a frequency structure that is impossible in a natural sound. (C) Semantic processing (SP) test: the patient must judge whether sound pairs from a range of human, animal and environmental sounds were generated by the ‘same’ source (such as a horse neighing and horse galloping; top panel) or by a ‘different’ source (such as a cat mewing and human male snoring; bottom panel). More information on the PPP, APP and SP tests is given in ^(‎‎17)^.
